# An *in vitro* correlation of metastatic capacity and dual mechanostimulation

**DOI:** 10.1371/journal.pone.0207490

**Published:** 2018-11-14

**Authors:** Indrajyoti Indra, Alexander N. Gasparski, Karen A. Beningo

**Affiliations:** Department of Biological Sciences, Wayne State University, Detroit, Michigan, United States of America; University of Texas MD Anderson Cancer Center, UNITED STATES

## Abstract

Cells are under the influence of multiple forms of mechanical stimulation *in vivo*. For example, a cell is subjected to mechanical forces from tissue stiffness, shear and tensile stress and transient applied strain. Significant progress has been made in understanding the cellular mechanotransduction mechanisms in response to a single mechanical parameter. However, our knowledge of how a cell responds to multiple mechanical inputs is currently limited. In this study, we have tested the cellular response to the simultaneous application of two mechanical inputs: substrate compliance and transient tugging. Our results suggest that cells within a multicellular spheroid will restrict their response to a single mechanical input at a time and when provided with two mechanical inputs simultaneously, one will dominate. In normal and non-metastatic mammary epithelial cells, we found that they respond to applied stimulation and will override substrate compliance cues in favor of the applied mechanical stimulus. Surprisingly, however, metastatic mammary epithelial cells remain non-responsive to both mechanical cues. Our results suggest that, within our assay system, metastatic progression may involve the down-regulation of multiple mechanotransduction pathways.

## Introduction

Mechanotransduction is a mechanism that regulates cellular behaviors during development [1, 2] tissue morphogenesis [3, 4], wound healing [5], and cancer cell invasion [6, 7]. A number of molecular players involving in mechanotransduction have been identified, including integrins [8], stretch-activated ion channels [9], cadherins [10] and focal adhesion kinases [11]. With the assistance of other signaling molecules working in concert, mechanical signals are converted into molecular responses. Examples of these responses include actin polymerization, integrin activation, tyrosine phosphorylation and the secretion of signaling molecules for survival, adhesion, proliferation and cell migration [12–14]. Nonetheless these responses arise upon the application of a single mechanical stimulus. Our understanding of what occurs to these mechanotransduction responses when multiple mechanical stimuli are applied simultaneously is currently limited. However, it has previously been reported that endothelial cell migration is positively influenced when fluid shear stress is applied to cells on compliant substrates, but not on harder substrates [15]. Additionally, a recent study found increased nitric oxide synthase activity when arterial endothelial cells were cultured on compliant substrates with applied laminar shear stress [16]. Aside from these studies, little else is known of how multiple mechanical cues are interpreted, particularly in the context of normal verses metastatic cells.

Cells *in vivo* are exposed to complex biophysical cues which play important roles in tissue patterning, development and individual cell behavior [17–20]. The way individual cells within a tissue respond to these extracellular cues and maintain the tissue architecture is largely unknown. It is believed that cellular behavior changes to accommodate the differences in extracellular biophysical cues that occur during differentiation and development. For example, the tissue repair process is concomitant with the stiffening of the tissue. The change in compliance results from extracellular matrix (ECM) synthesis and a pulling force that are exerted by the contractile myofibroblast. These factors work to close the wounded edges [21]. Similarly, mammary gland development involves the deposition of ECM and an accumulation of stromal fibroblasts for the formation of the ductal tree [22]. However, abnormal stiffening of the tissue and excessive contractile force result in fibrosis during wound healing and tumor formation in the breast [23, 24]. Given the importance of multiple mechanical cues in maintaining tissue integrity, it is necessary to understand the cellular response when more than a single mechanical input is received in both normal and disease contexts.

We previously showed that in mammary epithelial cells, the gain of metastatic capacity leads to a decrease in compliance sensing [25]. We tested those same cell lines in this two-dimensional assay system to determine if metastatic progression correlates in a loss of mechanosensing. The three murine breast cancer cell lines (67NR, 168FARN and 66cl4) originated from a single parental breast tumor, but each has a different capacity to move through the classical metastatic cascade. Briefly, 67NR is non-metastatic and can only form primary tumors whereas 168FARN can invade and enter the vasculature but cannot form secondary tumors. On the other hand, 66cl4 can complete all steps of the metastatic cascade required for the formation of secondary tumors [26].

Other studies have shown that the cellular response to substrate compliance [27, 28] or tugging forces [29, 30] are cell type dependent. In this study, we developed a novel two-dimensional *in vitro* assay system to understand how cells respond to substrate compliance and transient tugging forces, simultaneously. Substrate compliance is varied with two adjacent polyacrylamide hydrogels of a hard and soft stiffness that are physiologically relevant to the tumor microenvironment. Transient tugging forces are produced using a single magnetic bead embedded within the gel above a rotating magnet. As the magnet below rotates, it produces a ‘tugging’ force towards one of the two adjacent hydrogels because the bead is polymerized within the gel. We found that normal and non-metastatic mammary epithelial cells respond differently to dual mechanical inputs in comparison to metastatic mammary epithelial cells. When both mechanical cues are provided within the two-dimensional system, normal and non-metastatic cells preferentially responded to transiently applied mechanical cues by overriding the mechanical signal from the substrate compliance. Surprisingly, metastatic tumor cells did not respond to either of these mechanical cues. We interpret this to suggest that metastatic progression could be associated with the down regulation of select mechanosensors leading to reduced mechanotransduction.

## Materials and methods

### Cell culture

Four sub-populations of murine breast cancer cell lines derived from the same primary tumor, but possessing variable metastatic potential (a generous gift from Dr. Fred Miller, Karmanos Cancer Institute, Detroit, MI), and a normal murine mammary gland cell line (NmuMg) purchased from ATCC were used for this study. All cells are adherent and are able to form spheroids using the method described below. Mouse embryonic fibroblasts (MEFs) were purchased from ATCC. Cultures were maintained in Dulbecco’s Modified Eagle’s Medium (DMEM) containing 10% fetal bovine serum (Hyclone), and supplemented with 100U/mL penicillin, 2mM L-glutamine, and 100μg/mL streptomycin (Gibco). Cells were grown in a standard cell culture incubator at 37°C with 5% CO_2_.

### 3D spheroid preparation

Multicellular 3D spheroids were prepared by culturing cells on agar coated 96-well plates. Briefly, 96-well plates are coated with 50 μL of sterile 2% agar and UV sterilized for 30 minutes. Trypsinized cells were resuspended in cell culture media and approximately 1 X 10^4^ cells/mL were pipetted into each well. For spheroid development, the plate was placed on a rotating platform rotating at 1.83 Hertz inside the cell culture incubator until rounded spheroids formed. The spheroids were kept in culture until ready to use to allow them to proliferate to a suitable compactness and size.

### Substrate preparation

Polyacrylamide gels were prepared with a few modifications as described previously [31, 32]. The flexibility of the substrate was manipulated by maintaining the total acrylamide concentration at 5% while varying the bis-acrylamide concentration between 0.04% (Young’s modulus: 330 Pa, referred to as “soft”) and 0.1% (Young’s modulus: 1980 Pa, referred to as “hard”) [33]. Each substrate was embedded with 50μL of fluorescently labeled beads (0.2 μm carboxylated microspheres). To create the modified culture well, a 20mm hole was drilled with 1mm thickness through the bottom of a 60mm culture dish (Nunclon). A chemically treated coverslip [31] was then attached via vacuum grease to the bottom of the culture dish. Approximately, 250μL of hard substrate solution treated with ammonium per sulfate (APS) and tetramethylethylenediamine (TEMED) was pipetted into the culture well filling half of the well volume. A silanized square coverslip (25 x 25 μm) was placed on top of the solution leaving a gap on the opposite side of the well while the hard substrate polymerized for several minutes. Next, 250μL of APS- and TEMED-treated soft substrate was pipetted into the opposite half of the well. A paramagnetic bead of 800μm diameter (Cospheric) was quickly placed into the unpolymerized soft substrate using fine-tipped tweezers and positioned approximately 0.5–1 mm away from the border of the two substrates. The top coverslip was gently moved over the top of the unpolymerized substrate to close the gap. Following polymerization, the top coverslip was carefully removed. To facilitate cell adhesion, bovine plasma fibronectin (Sigma) at a concentration of 5μg/cm^2^ was conjugated on top of the polyacrylamide substrate as described previously [31]. After overnight incubation with fibronectin in 4°C, the substrates were rinsed with 1X PBS twice and UV sterilized for 30 minutes.

### Spheroid placement

After UV sterilization, 500uL of culture media was pipetted onto the substrate and placed into a cell culture incubator for 10 minutes to allow for temperature equilibration. A uniformly circular spheroid was selected and gently removed from the 96 well plate using a cut, sterile pipette tip. The spheroid was allowed to settle to the bottom of the cut tip via gravity and the tip was then gently transferred to the border between the hard and soft substrates. The spheroid was positioned on the border using a fine micropipette tip and allowed to attach for a period of 1–5 hours in a minimal amount of culture media inside the humidified incubator to prevent drying. Once attached, 4mL of culture media was gently added to the plate.

### Application of the mechanical stimulus

Mechanical stimulation was applied as described previously [32] with slight modification. Briefly, the assay plate was positioned 0.5 cm above a rare-earth magnet of 12,000 Gauss (25mm in diameter and 5.5mm in thickness). The magnet was rotated below the culture plate at 160 rpm (2.6 Hz) in an orbital field of 2cm on an orbital rotator (Barnstead, Roto Mix Type 50800) for 36 hours. The distance of the assay plate and the rotational speed of the magnet were adjusted based on the data obtained from bead displacements previously observed from cultured fibroblasts (as described in the results section).

### Measurement of cell dissemination

An Olympus IX81 ZDC inverted microscope was used to acquire phase contrast images of the spheroid both before and 36 hours after mechanical stimulation was applied. Live spheroids were imaged at 37°C with 5% CO_2_ within a custom stage incubator. Images were captured using a 10x/0.25NA CP-Achromat lens and SPOT Boost EM-CCD-BT2000 camera (Diagnostic Instruments) driven by IPLab software (BD Biosciences). The distance that cells disseminated from the spheroid was measured by drawing a line from the spheroid edge to the furthest cell disseminated using ImageJ (NIH).

## Results

### Designing an assay for the simultaneous application of two mechanical stimuli

**Fig 1:**
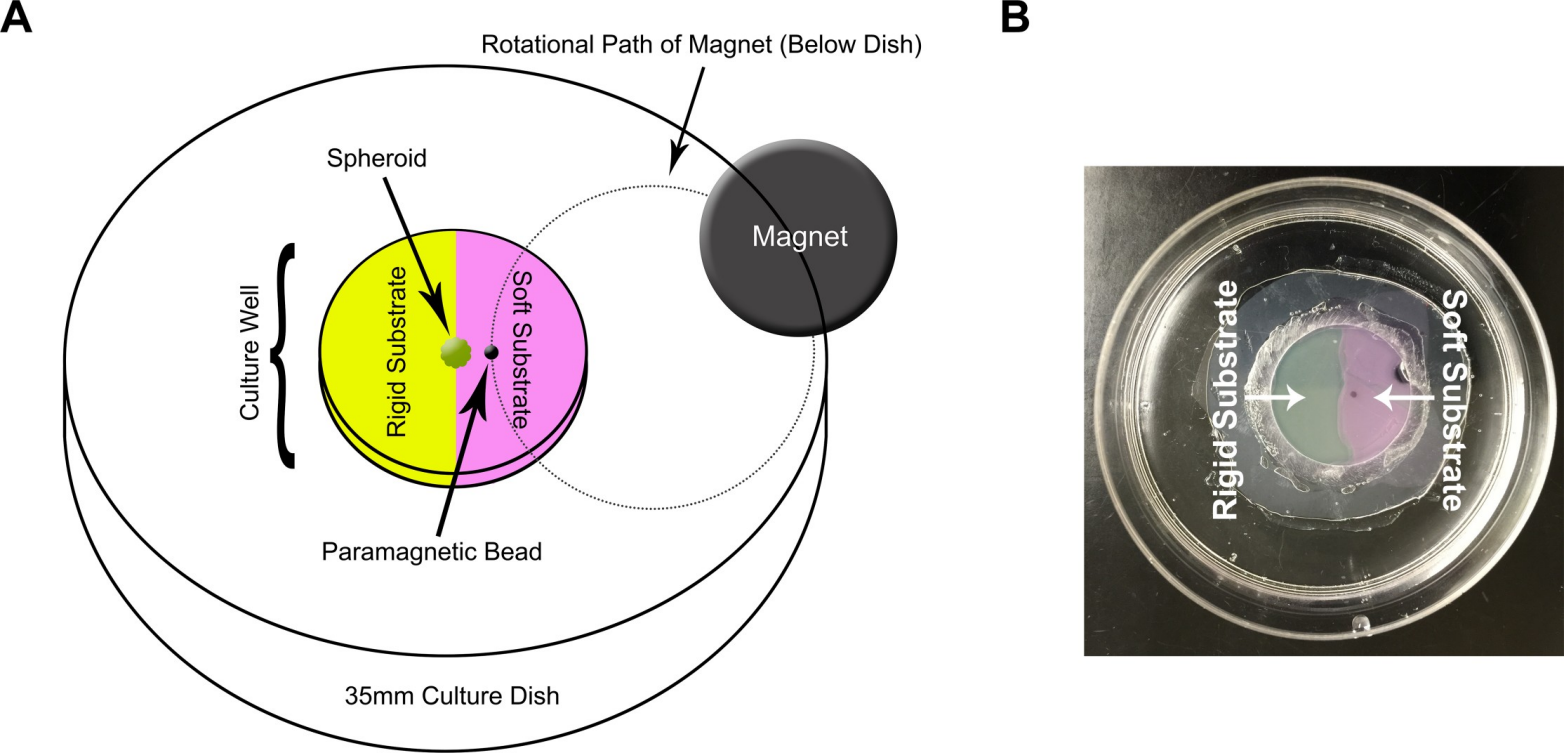
Substrate for an *in vitro* assay of dual mechanostimulation. (A) Schematic of the culture well cast with soft and hard polyacrylamide hydrogels side-by-side and conjugated with bovine plasma fibronectin on the surface. A paramagnetic bead was embedded within the soft substrate and positioned approximately 0.5–1 mm away from the border of the two substrates. A multicellular spheroid was placed on the border of the two substrates. A rare-earth magnet rotated 0.5 cm below the assay plate. The rotational path of the magnet is displayed as dotted lines. (B) The assay plate prior to the placement of a spheroid onto the substrate.

The purpose of this study was to understand how cellular sensing of substrate compliance and applied mechanical cues is affected with metastatic progression. To answer this question, we designed an *in vitro* assay system where both mechanical cues are simultaneously provided and manipulated. Variation in substrate compliance was produced by casting polyacrylamide hydrogels of two different stiffnesses side by side (Fig 1A and 1B). The entire substrate was conjugated with the extracellular protein fibronectin to create a uniform adhesive field for cell attachment. We chose fibronectin because we have previously shown that the compliance sensing properties of this cell line panel is fibronectin dependent [25]. To provide applied stimulation from the softer part of the substrate, an 800μm diameter paramagnetic bead was embedded within the soft hydrogel and positioned approximately 0.5–1 mm away from the border where the substrates of two different stiffnesses meet. Transient mechanical tugging was generated from the softer part of the substrate by placing the assay plate above a rotating rare earth magnet. The entire assay set-up was placed within a tissue culture incubator. The non-metastatic and metastatic breast cancer cell lines of the murine panel spheroids were placed at the border of the two compliances (hard and soft) and the applied stimulus was generated by the rotating magnet. For control conditions, plates were kept outside the magnetic field. Three-dimensional spheroids were used in this assay due to their physiological similarities to an *in vivo* tumor when considering intact cell-cell adhesions and dimensionality.

### Physiological relevance of transient stimulation

**Fig 2:**
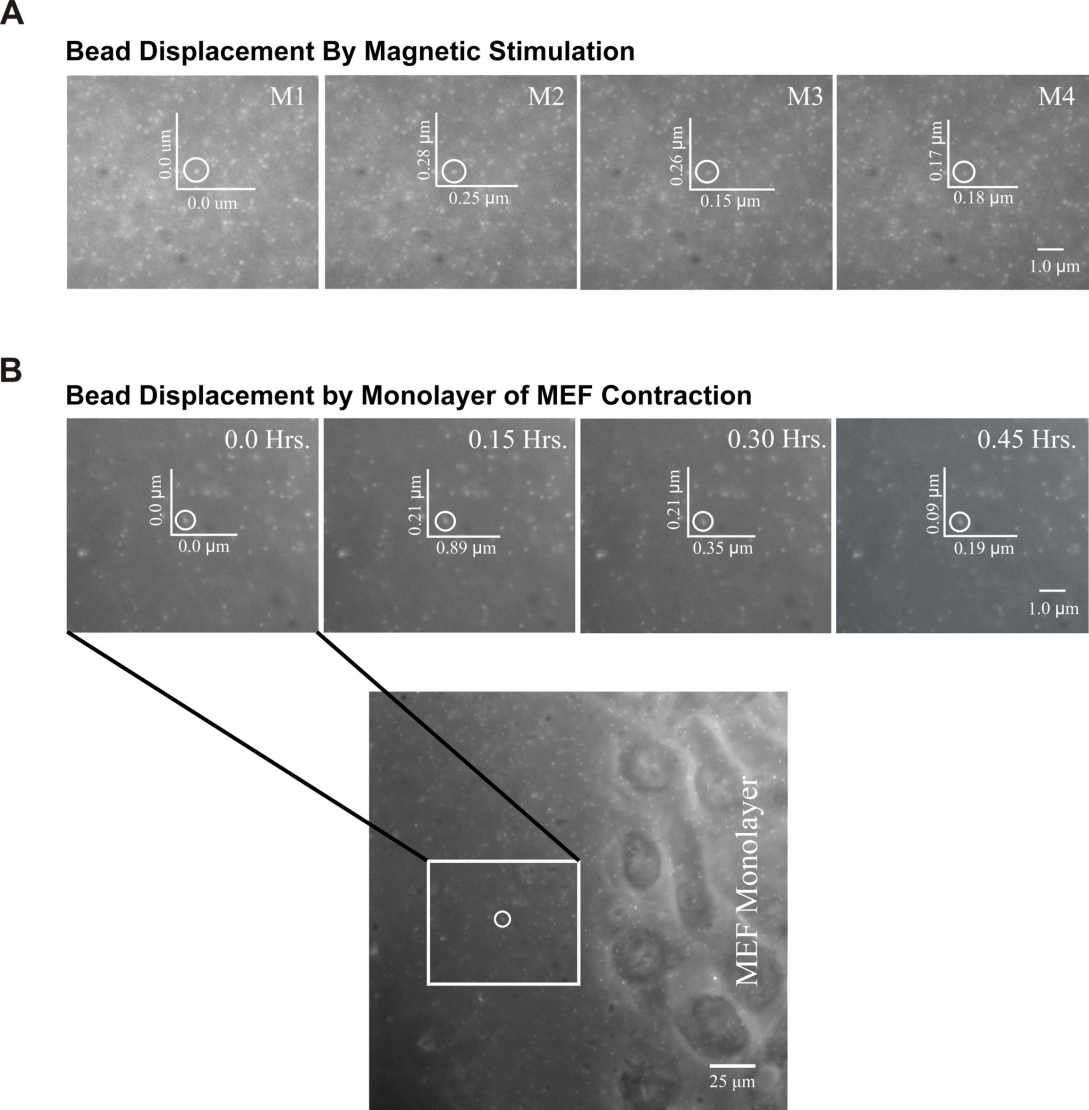
Bead displacement upon magnetic stimulation and cellular contraction. (A) The displacement of a fluorescent microbead (0.2μm) embedded within the assay substrate due to the pulling forces generated by the paramagnetic bead upon magnetic stimulation. X and Y coordinates of the fluorescent microbead are shown while the rotating magnet under the assay plate is on four equidistant positions of its rotational path (M1-M4). (B) Merged images of a monolayer of mouse embryonic fibroblast (MEF) cells and fluorescent microbeads (0.2μm) embedded within the assay substrate. A bead approximately 100 μm away from the edge of the cell monolayer was outlined and magnified (25X). The displacement of the bead is shown in X-Y coordinates on a 2D plane due to the contractile forces generated by MEF cells at 15 minute intervals. Images shown are representative from three different independent trials.

To understand the response of mammary epithelial cells to the contractile forces generated by neighboring cells, we adjusted the stimulation in our assay to be physiologically equivalent to fibroblasts. To do this, we first tested if the magnetic stimulation can transiently displace the paramagnetic bead embedded within the soft substrate (Fig 2A). Using a Gaussmeter, we determined that a 12,000 Gauss rare earth magnet of 25 mm in diameter and 5.5mm in thickness is capable of producing approximately 800 Gauss magnetic force if it rotates 0.5 cm beneath the assay plate at a speed of 2.33 Hertz and completes a 2 cm orbital diameter. We then wanted to know the magnitude of the force generated by this magnet on the substrate in order to determine if it is physiologically similar to the forces produced by cells. To do this, we simulated the assay under the microscope and observed a 0.15–0.25μm and 0.17–0.28μm displacement of embedded fluorescent microbeads in the x and y plane, respectively, due to the transient tugging force caused by the paramagnetic bead under magnetic stimulation. To determine if this pulling force is physiologically relevant, a monolayer of mouse embryonic fibroblast (MEF) cells was cultured on the assay substrate and the displacement of the embedded fluorescent microbead at the edge of cell monolayer was recorded (Fig 2B). The fluorescent bead displacement due to the contractile forces produced by MEF cells was found to be in the range of 0.19–0.89μm and 0.09–0.21μm in x and y planes, respectively. This indicated that forces generated by the cells is comparatively higher than that of the transient mechanical pulling caused by magnetic stimulation in our assay. Furthermore, the data obtained using our *in vitro* assay system is predicted to be more conservative than the *in vivo* situation because highly contractile myofibroblast cells found in mammary gland associated stroma is reported to produce higher contractile forces compared to the MEF fibroblasts [34].

### Transient mechanosensing overrides the compliance sensing properties in normal mammary epithelial cells

**Fig 3:**
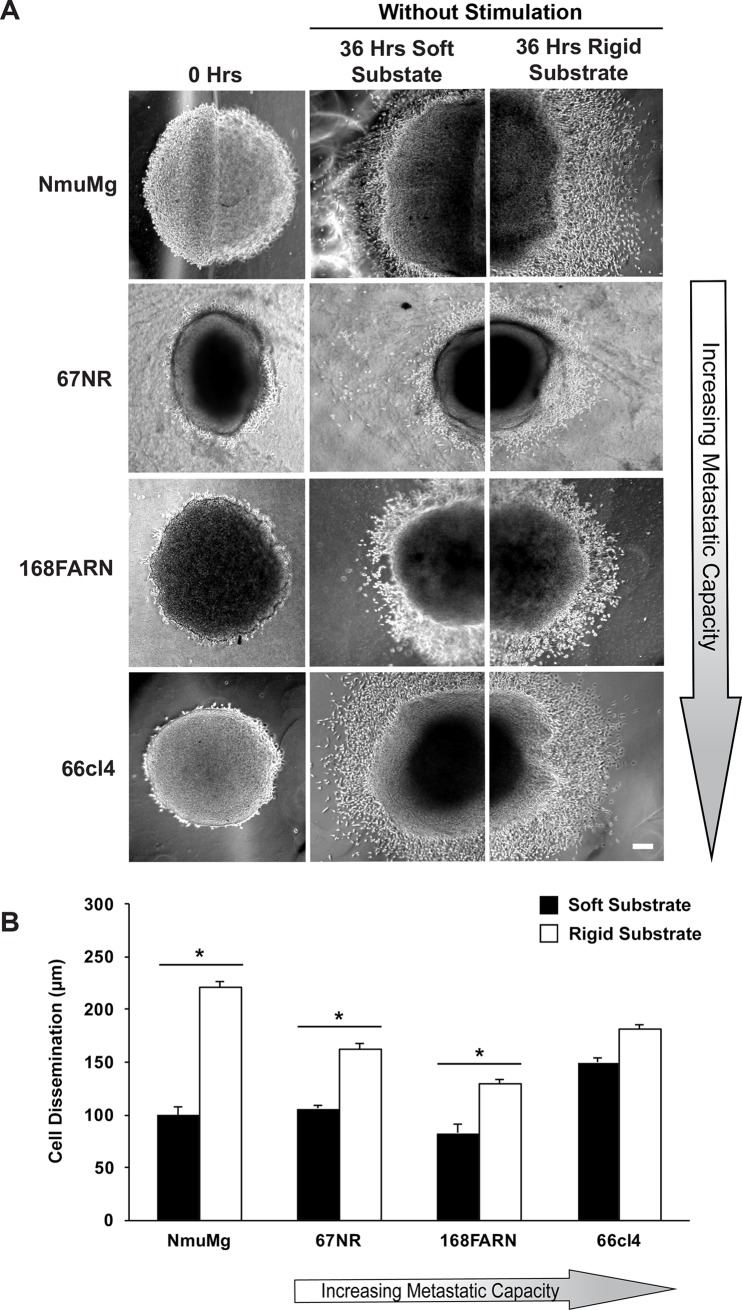
Compliance guided cellular dissemination is lost with metastatic progression. (A) Dissemination of cells from the multicellular spheroids without transient stimulation at 0 hours (left column) and 36 hours on soft (middle column) and hard (right column) substrate are shown for NmuMg and the panel of murine breast cancer cell lines. Scale bar: 100 μm. (B) Bar graph representing the length of disseminated cells from the edge of the spheroid after 36 hours with stimulation. Black and white bars represent the distance of disseminated cells (in μm) on soft and hard substrates, respectively. Error bars represent the mean ± s.e.m. from at least three separate experiments. *P<0.05 by the Student’s t-test.

**Fig 4:**
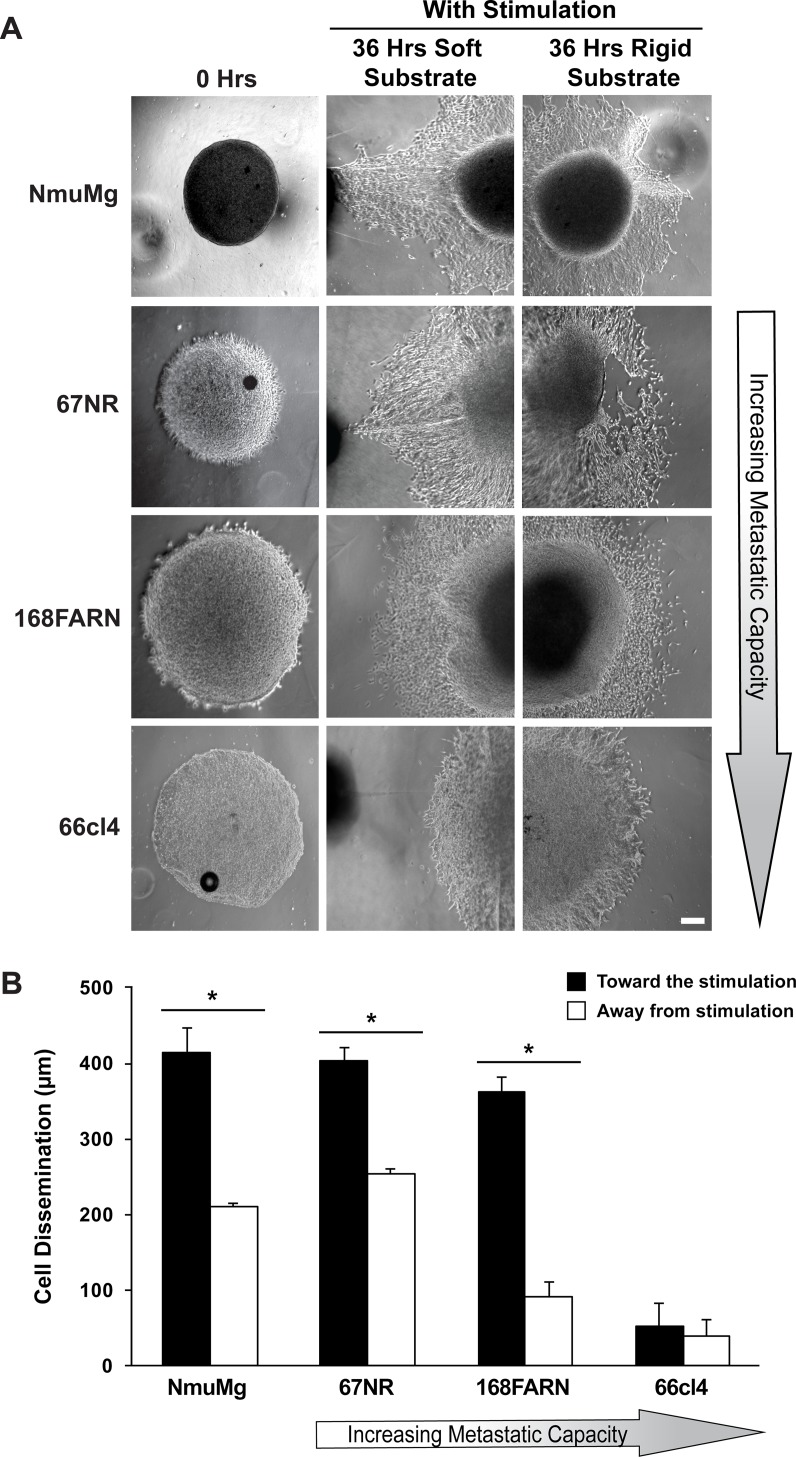
Normal and non-metastatic cells, but not metastatic cells, sense transient stimulation and override substrate compliance signaling. (A) Dissemination of cells from the multicellular spheroids with transient stimulation at 0 hours (left column) and 36 hours on soft (middle column) and hard (right column) substrates. Scale bar: 100 μm. (B) Bar graph representing the length of disseminated cells from the edge of the spheroid after 36 hours of stimulation. Black and white bars representing the distance of disseminated cells (in μm) towards (on soft substrate) and away (on hard substrate) from the stimulation, respectively. Error bars represent the mean ± s.e.m. from at least three separate experiments. *P<0.05. by the Student’s t-test.

Under normal physiological conditions, mammary epithelial cells encounter changes in substrate compliance and transient pulling forces [22]. Based on this, we first tested the response of normal murine mammary gland cells (NmuMg) in our assay system (Figs 3A and 4A). For this purpose, a multicellular spheroid of NmuMg was placed at the border of two compliant hydrogels conjugated with fibronectin. After adhesion of the spheroid to the substrate, the assay plate was kept with or without constant magnetic stimulation for 36 hours. Microscopic images of cellular dissemination were taken before and after stimulation. The distance of cell dissemination out of the spheroid was calculated by drawing a line from the edge of the spheroid to cells that had disseminated furthest from the spheroid and plotted as a bar graph. Similar to our previous finding [25], the dissemination of NmuMg cells was found to be dependent on the stiffness of the substrate. When transient stimulation was not provided, the edge of the disseminating NmuMg cells extended to 221 μm on the harder half of the substrate, in contrast to a distance of 100 μm on the softer substrate (*P*<0.05) (Fig 3B). Surprisingly, a transition in the pattern of cellular dissemination was observed when transient tugging forces were applied. The dissemination of NmuMg cells from the spheroid was now 414 μm on the soft part of the substrate containing the magnetic stimulation as compared to 211 μm on hard part of the substrate without magnetic stimulation (*P*<0.05) (Fig 4B). This suggests that transient tugging forces might override the compliance sensing mechanism in normal mammary gland cells because they have a lower dissemination distance on the softer substrate until stimulation is applied.

### Metastatic progression correlates with an inability to sense both compliance and transient mechanical stimulation

We have previously shown that the ability of mammary epithelial cells to sense changes in compliance decreases with the gain of metastatic capacity [25]. This led us to ask whether metastatic progression is also associated with a progressive loss in the ability to sense transient tugging forces. We tested the panel of murine breast cancer cell lines (67NR, 168FARN and 66cl4) of varying metastatic potential in our assay system. Without transient stimulation, 67NR and 168FARN cells disseminated 56 and 46 μm further, respectively, on the hard substrate compared to the softer substrate (*P*<0.05) (Fig 3B). This result demonstrates that, as with NmuMg cells, 67NR and 168FARN cells responded to the changes in substrate compliance. Furthermore, as we have reported earlier [25], a gradual decline in the response to substrate compliance was observed with metastatic progression (Fig 3A and 3B). However, the preferential dissemination toward the more hard substrate is lost when we tested the most metastatic cell line, 66cl4 (Fig 3A and 3B), which is in agreement with our previous finding [25]. When we provided transient stimulation, like normal mammary gland cells, the non-metastatic cell lines (67NR and 168FARN) responded to the transient stimulation by overriding the substrate compliance stimulus and disseminated onto the soft substrate (Fig 4A). The 67NR and 168FARN cell lines disseminated by 150 and 271 μm further in distance, respectively, on the softer substrate compared to the hard substrate (*P*<0.05) (Fig 4B). However, the metastatic cell line, 66cl4, neither sensed changes in compliance nor did it respond to the transient stimulation (Fig 4B). Together these results suggest that the cells within this panel may lose their mechanosensing abilities for both compliance and transient tugging and pulling as they progress in metastatic capacity.

## Discussion

The importance of mechanical forces in regulating the cellular behavior has been well established [35–37]. However, many of these studies are concentrated on understanding the cellular behavior in response to a single type of mechanical force. To advance our understanding of how mechanical cues affect *in vivo* systems where multiple biophysical cues are present simultaneously, we must also study cell behavior in response to complex mechanical environments. In this study, we have simultaneously provided two forms of mechanical stimulation (compliance and transient tugging) and asked whether cells can respond to these mechanical cues when delivered simultaneously. We have also correlated these mechanosensory responses to metastatic progression.

Given the importance of compliance and transient mechanical forces in mammary gland development and tumor progression, we have tested the influence of these mechanical inputs on normal murine mammary epithelial cells and a panel of murine breast cancer cells. Our assay system was designed to provide both simplicity and physiological relevance to determine the cellular response to more than a single form of mechanical cue. Compliance of the soft and hard substrates were optimized based on the physiological range of compliance reported during breast tumor formation (0.2 kPa to 2.0 kPa) [38, 39]. The magnitude of transient stimulation provided in our assay system was also optimized based on the contractile forces generated by a monolayer of fibroblast cells. In addition, to understand the cellular response in a tissue context, we have tested the mechanical cues on multicellular spheroids instead of individual cells.

We have previously shown that the ability to sense changes in compliance decreases gradually as cells become more metastatic [25]. However, in this current study, when the transient mechanical stimulation was provided along with the compliance cue, the normal and non-metastatic (67NR and 168FARN) cells responded to this applied cue by ignoring the substrate compliance. Surprisingly, highly metastatic 66cl4 cells did not show any change in dissemination, as if the necessary mechanotransduction pathway was turned off. The inability to sense these mechanical cues could result in the loss of directional migration in highly metastatic cells, as previously described for cancer cell invasion and metastasis [40, 41]. From these results, we surmise that transient stimulation likely overrides the ability to sense changes in compliance and unlike metastatic cells, tumorigenic, but non-metastatic cells, retain normal sensing behavior. In addition, we suggest that mammary epithelial cells respond to one mechanical input at a time, but the dominance of a particular type of mechanical cue might be cell type dependent. However, we speculate that a cell can respond to multiple mechanical stimuli when other combinations of biophysical cues are provided. It is also likely that any number of biochemical factors would influence these observations, as the biophysical and biochemical contributions to cellular physiology are not independent.

Our simple assay system provides an *in vitro* methodology for the simultaneous application of more than one mechanical cue. In the future, it will be important to determine the cellular response to different combinations of multiple mechanical cues using *in vitro* and *in vivo* approaches. Furthermore, the mechanosensors and the mechanotransduction pathways involved in sensing the transient stimulation and how this pathway is influenced by metastatic progression will need to be examined. We have previously shown that integrin β1 activation and phosphorylation of focal adhesion kinase (FAK) at tyrosine 397 is involved in sensing the substrate compliance [25]. Based on our observation of increased dissemination and migration of normal and non-metastatic cells on a softer substrate, we would reason that in the presence of transient stimulation, the sensing of cellular compliance is turned off by over activation of β1 integrin and subsequent increased FAK Y^397^ phosphorylation. Thus, it is reasonable to examine the status of the active form of integrin β1 and Y^397^ FAK in cells disseminated on softer substrates in response to applied stimulation. It is also possible that other mechanosensory molecules could be involved in sensing the transient stimulation and their activation could result in over activation of β1 integrin, increased FAK phosphorylation and down-regulated compliance sensing. In addition, the β3 subunit of integrin has been shown to be a mechanosensor [42, 43], hence the next logical step would be to determine the localization and activation of β3 integrin in response to transient stimulation. Other non-integrin molecules may also be at play, such as the calpain family of proteases. In highly metastatic breast cancer cells, it was found that calpain-2 plays an important role in the cleavage of talin and focal adhesion turnover, which affects the migration of breast cancer cells in stiff tumor environments [44].

Since a cell has a wide array of canonical and non-canonical mechanosensors at their disposal, it should be of no surprise that a cell makes a ‘judgment’ when presented with multiple, sometimes conflicting, mechanical stimuli. This is likely a very complex process that widely varies between cells types, especially among cancers. Thus, an understanding of how a cell reacts to a multitude of competing mechanical stimuli within the tumor microenvironment can provide greater insight into the metastatic process.
